# Identification of CXCR4 Upregulation in Diffuse Large B-Cell Lymphoma Associated with Prognostic Significance and Clinicopathological Characteristics

**DOI:** 10.1155/2022/3276925

**Published:** 2022-06-21

**Authors:** Yi-an Zhang, Xue Yang, Jiamei Yao, Yuhong Ren, Peng Liu

**Affiliations:** ^1^Department of Hematology, Zhongshan Hospital, Fudan University, Shanghai, China; ^2^Department of Pathology, Zhongshan Hospital, Fudan University, Shanghai, China; ^3^Cancer Center, Zhongshan Hospital, Fudan University, Shanghai, China

## Abstract

**Background:**

Diffuse large B-cell lymphoma (DLBCL) is a heterogeneous malignant lymphoma with distinct characteristics. Patients with treatment failure after the standard immunochemotherapy have worse prognosis, which implies the necessity to uncover novel targets. The C-X-C chemokine receptor 4 (CXCR4) overexpression has been identified in several hematopoietic malignancies. However, the expression signatures and prognostic significance of CXCR4 in DLBCL associated with clinicopathological features remain unclear.

**Methods:**

Gene expression profiles of DLBCL were obtained from the Cancer Genome Atlas (TCGA) and Gene Expression Omnibus (GEO) databases. Then, a meta-analysis with an integrated bioinformatic analysis was performed to assess the relationship between CXCR4 expression and clinicopathological features of DLBCL. Finally, experimental verification including immunohistochemical (IHC) staining and real-time quantitative PCR (qPCR) was carried out using patient samples. In vitro cell line viability tests were conducted using CXCR4 inhibitor WZ811.

**Results:**

DLBCL patients with activated B-cell-like (ABC) subtype have higher expression level of CXCR4 with worse survival. Differential expressed genes in the CXCR4-upregulation group were enriched in canonical pathways associated with oncogenesis. DLBCL with CXCR4 upregulation had lower degree of CD8^+^ T cell infiltration. TIMER analysis demonstrated that the CXCR4 expression was positively correlated with the expression of CD5, MYC, NOTCH1, PDCD1, CD274, mTOR, FOXO1, and hnRNPA2B1 in DLBCL. IHC study in patient samples showed the positive correlation between CXCR4 and nongerminal center B-cell (non-GCB) subtype and mTOR expression. Meanwhile, quantitative polymerase chain reaction results revealed that high CXCR4 mRNA level was correlated to double-hit DLBCL. Finally, cell viability test showed that WZ811 exerted antiproliferation effect in DLBCL cell lines in a dose-dependent manner.

**Conclusion:**

CXCR4 was upregulated in ABC-DLBCL associated with worse prognosis. Our analysis predicted CXCR4 as a potential target for DLBCL treatment, which may serve as an inhibitor both on BCR signaling and nuclear export warranting further investigation in clinical trials.

## 1. Introduction

Diffuse large B-cell lymphoma (DLBCL) is one of the most common types of non-Hodgkin lymphoma (NHL), which is considered aggressive but potentially curable. Despite 60% patients can be cured with the standard immunochemotherapy of R-CHOP (rituximab plus cyclophosphamide, doxorubicin, vincristine and prednisolone), those with treatment failure after R-CHOP often have worse outcomes [1]. With the advance in second-generation sequencing to uncover novel molecular biomarkers and signaling pathways, there is a trend toward using XR-CHOP treatment, namely, adding new drugs to the standard R-CHOP regimen. For instance, bortezomib, lenalidomide, ibrutinib, BCL2, and EZH2 inhibitors, as well as inhibitors of PI3K/AKT/mTOR, have now being used as first-line therapy in combination with R-CHOP in patients with estimated poor prognosis according to pathological subtypes in several biomarker-driven clinical studies [2]. Nevertheless, there is limited evidence that DLBCL patients benefit more from XR-CHOP than R-CHOP [3, 4]. Therefore, further research on the genetics and pathogenesis of DLBCL is needed to identify novel molecular targets and therapeutic vulnerabilities.

Chemokines and their receptors play an important role in oncogenesis, progression, and dissemination of tumor cells [5]. CXCR4 is a chemokine receptor involved in a series of biological processes including hematopoeisis and immune response, which is highly expressed in a variety of hematological malignancies [6]. CXCR4 is a prognostic biomarker in lots of cancer diseases such as gastrointestinal cancer [7], breast cancer [8], and lung cancer [9] [10, 11]. Recent studies revealed that the CXCR4 and its ligand CXCL12 are linked to the pathogenesis of lymphohematopoietic malignancies [12, 13]. The CXCR4 expression was reported to be associated with poor prognosis, and the inhibition of CXCR4 renders tumor cells more sensitive to chemotherapy [14].

To date, there has been no consensus on the correlation between the prognostic significance of CXCR4 and clinicopathological characteristics of DLBCL. In a large cohort (training/validation cohort of 468/275), the increased expression of CXCR4 was associated with the activated B-cell-like (ABC) subtype in patients with newly diagnosed DLBCL. However, it was also an independent risk factor for progression-free survival (PFS) in germinal center B-cell-like- (GCB-) DLBCL [15]. In another study by et al., CXCR4 was not associated with the prognosis of DLBCL patients [16]. Differed results seem controversial, and an integrated analysis is lacking. In this study, we used a meta-analysis and an integrated bioinformatics analysis to conduct a comprehensive assessment of the relationship between CXCR4 expression and DLBCL. Results were further verified by immunohistochemical (IHC) staining on tissue samples from independent cohorts with in vitro cell function tests.

## 2. Materials and Methods

### 2.1. Data Mining and Clinical Samples

We searched DLBCL gene expression datasets from the Cancer Genome Atlas (TCGA) database (https://portal.gdc.cancer.gov/) and the Gene Expression Omnibus (GEO) database (https://www.ncbi.nlm.nih.gov/geo/). By using“DLBCL” or “diffuselargeBcell lymphoma,” “homo sapiens,” and “expression profiling” as qualifiers, we searched and screened “microarray” and “RNA sequencing” data from the year 2006-2018 in the GEO database. The inclusion criteria were as follows: detectable CXCR4 expression level, available clinicopathological data, number of datasets larger than 30 patients, and tissue samples. The diagnosis and classification of DLBCL cases were based on the criteria of the 2008 World Health Organization classification of the lymphoid neoplasms. Two independent validation cohorts, one is tissue microarray including forty-four de novo DLBCL patients, another is formalin-fixed paraffin-embedded (FFPE) samples of 59 de novo DLBCL patients, were both obtained from Zhongshan Hospital, Fudan University. This study was approved by the Institutional Review Board (IRB) of Zhongshan Hospital Affiliated to Fudan University (B2021-025R) and was conducted in accordance with the principles of the Declaration of Helsinki. Written informed consent was signed by each participant.

### 2.2. Integrated Bioinformatic Analysis

Databases from GPL570 (GSE11318, GSE23501, GSE19246, GSE31312, GSE53786, GSE64555, GSE56313, GSE74266, and GSE93986) were selected for gene set enrichment analysis (GSEA), protein-protein interaction (PPI) network, and immune cell infiltration (ICI) analysis. Subjects were categorized into low and high expression groups according to their median CXCR4 expression level. Normalization was performed, and batch effects were eliminated using the “SVA” and “limma *R*” packages (version3.12, http://www.bioconductor.org), and an adjusted *P* < 0.05 was selected as the threshold for enriched terms. Genes with |log2FC (fold change) >2 were considered differentially expressed. To explore the key signal pathway differences between the CXCR4 high and low expression population, genes were characterized using the GSEA 4.1.0 software (https://www.broadinstitute.org/gsea/). Datasets c2.all.v7.2.symbols.gmt (C2: curated gene sets) and c5.all.v7.2.symbols.gmt (C5: ontology gene sets) were obtained from the MsigDB database on the GSEA website (http://www.gsea-msigdb.org/), and the number of permutations was set at 1000. The gene sets were normalized using an enrichment score of >1, with *P* < 0.05. A false discovery rate (FDR) in GSEA of <0.25 was considered as significant enrichment gene set and was used as the standard for distinguishing significant enrichment signaling pathways. The protein-protein interaction (PPI) network was analyzed using the STRING website (http://string.embl.de/). Pathway analysis of 30 hub genes was further analyzed using the online tool KOBAS 3.0 (http://kobas.cbi.pku.edu.cn/kobas3).

### 2.3. ICI Level and Tumor Immune Estimate Resource (TIMER) Analysis

The CIBERSORT algorithm was performed to evaluate ICI in DLBCL tissues between CXCR4 high and low expression group. This algorithm transforms the normalized gene expression matrix into the composition of infiltrating immune cells. Analysis of ICI level between CXCR4 high and low expression groups was conducted by the “CIBERSORT *R*” package. Then, samples selected with a CIBERSORT *P* value <0.05 was filtered and reserved for the following analysis. We also used online tool TIMER2.0 (http://timer.cistrome.org/) to conduct Kaplan-Meier analysis to explore the infiltration of various immune cell subsets in patient survival from the TCGA database. Then, we used the TCGA database to explore how the CXCR4 expression was correlated with the expression of biomarkers that showed significance in the GSEA analysis.

### 2.4. Immunohistochemistry and Evaluation Criterion

An independent cohort of 44 de novo DLBCL tissue microarrays from Zhongshan Hospital was stained with eight antibodies. IHC staining was performed on the Leica platform (Bond Max) according to the antibody manual (Table S1) and manufacturer's instructions. IHC was assessed by two pathologists independently. They reexamined the immunostained slides to reach a consensus when there were inconsistencies. The percentage of positive CXCR4 stained cell area was scored into four grades: 0 for none; 1, <25%; 2, 25% to 50%; 3, 50% to 75%; and 4, >75%. The intensity was as follows: 0, negative; 1, weakly staining; 2, moderately staining; and 3, strongly staining. The final immunoreactivity scores were obtained by multiplying the percentage and the intensity score (0 to 12), and a final score lower than 2 was defined as CXCR4 negative, while a score ≥ 2 represented CXCR4 positivity.

### 2.5. Real-Time Quantitative Polymerase Chain Reaction(qPCR)

Five 5 *μ*m unstained FFPE tissue sections were freshly cut for gene expression analysis. Total RNA was extracted using an FFPE Total RNA Isolation Kit (Canhelp Genomics Co., Ltd., Hangzhou, China) according to the protocols. For each specimen, reverse transcription was performed on isolated total RNA using the High-Capacity cDNA Reverse Transcription Kit with RNase Inhibitor (Applied Biosystems, Foster City, CA, USA). Next, the real-time PCR (RT-PCR) reaction was applied on the 7500 Real-Time PCR system (Applied Biosystems, Japan) for analyzing gene expression profiles. The RT-PCR program was initiated at 95°C for 10 minutes, followed by 40 cycles at 95°C for 15 seconds, and 60°C for 1 minute. The mRNA quantity of specific genes, calculated using the 2^-△△CT^ method, was normalized against GAPDH. All the measurements were performed in triplicate. The sequences of the CXCR4 primer pairs were as follows: CXCR4-F: 5′-GGTGGTCTATGTTGGCGTCT-3′, CXCR4-R: 5′-TGGAGTGTGACAGCTTGGAG-3′.

### 2.6. In Vitro Proliferation Assay

The effects of CXCR4 inhibitor WZ811 (MedChemExpress) were investigated by a Cell Counting Kit-8 (CCK-8; Dojindo, Kumamoto, Japan) assay. Human DLBCL cell line OCI-Ly3 (ABC type) and SU-DHL4 (GCB type) were purchased from Fuheng Cell Center, Shanghai. For time-dependent assay, tumor cells were plated in a 96-well plate, and the cell density was depended on cultured time. For dose-dependent assay, tumor cells were plated at a density of 3000 cells with different concentrations of WZ811. All assays were performed in quadruplicate. At 0 h, 2 h, and 24 h, the cell proliferation assay was performed according to the manufacturer's instructions. Absorbance was measured at a wavelength of 450 nm using a microplate reader (Flexstation III ROM V2.1.28, USA).

### 2.7. Statistical Analysis

Stata 14.2 (StataCorp, TX, USA) software was applied to conduct meta-analysis. Correlations between clinicopathological features and gene protein expression were determined using a *X*^2^ test. Study heterogeneity was accessed by a *X*^2^ test based on the *Q* statistical test. We also applied a sensitivity analysis to evaluate the stability of the pooled results. Univariate and multivariate Cox regression analyses were performed using *R* software (version 4.0.4). The effects of the CXCR4 expression on OS and PFS in DLBCL were analyzed using Kaplan-Meier analysis. The nonparametric Spearman *r* correlation coefficient was employed to assess the correlations between CXCR4 and other biomarkers. Cell viability test was analyzed using one-way ANOVA, and *P* < 0.05 was considered statistically significant.

## 3. Results

### 3.1. Correlations between CXCR4 Expression and DLBCL Subtypes Associated with Prognosis by Meta-Analysis

Information available for 2515 patients derived from 20 datasets were included in meta-analysis. The TCGA database (workflow type: RNASeq-FPKM) included 46 DLBCL cases with CXCR4 expression values and clinical characteristics (including gender, age, clinical stage, and survival). Nineteen datasets in the GEO database (experiment type: expression profiling by array) were included, with 15 datasets available for DLBCL subtypes and 10 datasets available for survival information (Table 1**)**. No heterogeneity was found across the datasets (*I*^2^ = 12.1%, *P* = 0.321).

**Figure 1:**
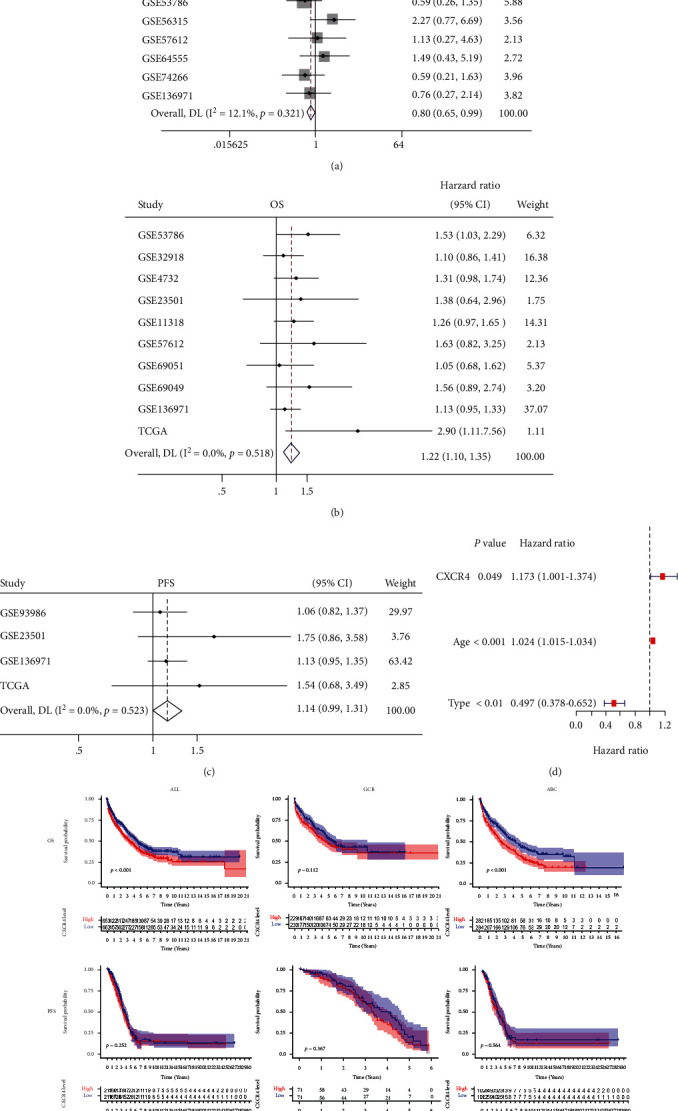
Forest plots of the (a) CXCR4 expression and cell of origin subtypes in DLBCL. (b) The CXCR4 expression and overall survival in DLBCL. (c) Progression-free survival. (d) Multivariate cox proportional hazards regression analysis. (e) Integrated analysis of the CXCR4 expression on overall survival (upper) and progression-free survival (lower) in DLBCL analyzed by Kaplan–Meier plot curves. Abbreviations: COO: cell of origin; overall survival: OS; progression-free survival: PFS; GCB: germinal center B-cell-like; ABC: activated B-cell-like.

14 datasets were analyzed available for DLBCL subtypes. Pooled results indicated that 5he CXCR4 expression was significantly higher in ABC subtype than in GCB subtype of DLBCL (odds ratio [OR] = 0.80, 95% CI: 0.65 to 0.99, *P* = 0.039) (Figure 1(a)). 10 datasets were analyzed available for survival data including overall survival (OS) and progression-free survival (PFS). The random effect model (*I*^2^ = 0.0%, *P* = 0.518) was used, and the pooled results indicated that the CXCR4 overexpression in DLBCL patients was related to poorer OS (HR = 1.22, 95% CI: 1.10 to 1.35, *P* < 0.001) (Figure 1(b)). After each exclusion, the impact on the combined data is small. The combined effect (random effect model, *I*^2^ = 0.0%, *P* = 0.523) showed that the CXCR4 expression in patients with DLBCL had no significant influence on PFS (HR = 1.14, 95% CI: 0.992 to1.307, *P* = 0.065) (Figure 1(c)). However, patients with higher CXCR4 expression tended to have poorer PFS.

To further clarify whether CXCR4 is an independent risk factor for DLBCL, we conducted univariate and multivariate analyses. Multivariate analysis result indicated that CXCR4 acts as an independent factor in the prediction of prognosis in DLBCL patients (Figure 1(d)). In the integrated analysis of survival (Figure 1(e)), a trend of association between CXCR4 high expression and unfavorable OS was found in DLBCLABC type (*P* < 0.001) but not GCB type (*P* = 0.252), and no association between CXCR4 high expression and unfavorable PFS for both types was found (*P* > 0.05).

### 3.2. Gene Enrichment Analysis Related to CXCR4 Expression by GSEA and PPI Network

**Figure 2:**
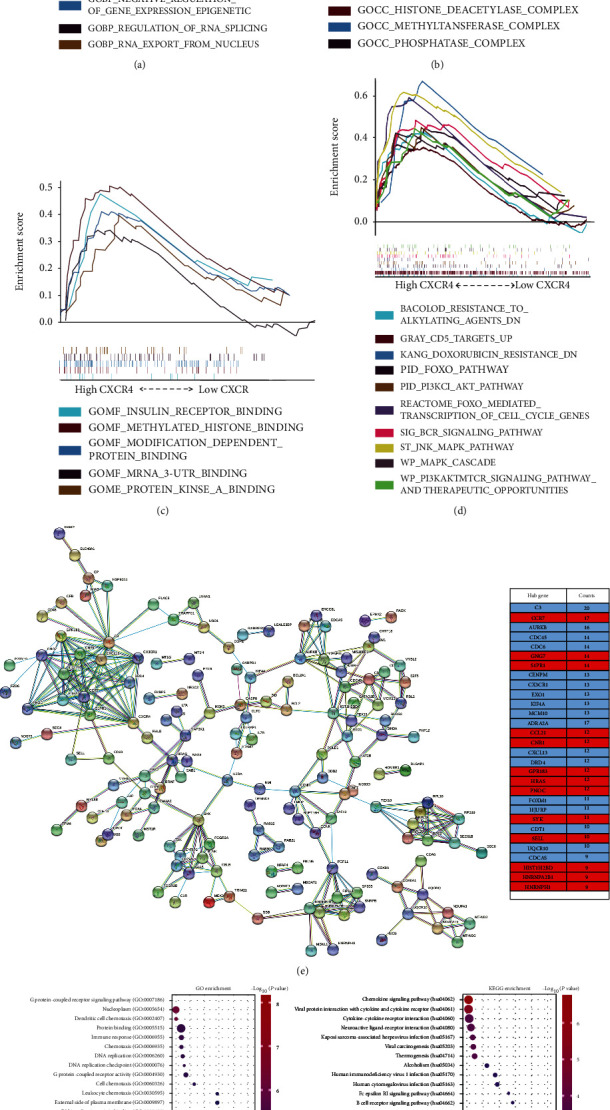
GSEA Gene Ontology (GO) analysis showed that CXCR4-related genes were enriched in (a) biological process (BP), (b) cellular component (cc), and (c) molecular function (MF). (d) GSEA C2 curated gene sets analysis. (e) Protein-protein interaction network of CXCR4 by the STRING website (left). Selected 30 hub genes (C3, CCR7, AURKB, CDC45, CDC6, GNG7, S1PR1, CENPM, CX3CR1, EXO1, KIF4A, MCM10, ADRA2A, CCL21, CNR1, CXCL13, DRD4, GPR183, HRAS, PNOC, FOXM1, HJURP, SYK, CDT1, SELL UQCR10, CDCA5, HIST1H2BD, HNRNPA2B1, HNRNPH1) as center nodes of the interaction network are shown in the right table (red: upregulation; blue: downregulation). (f) GO and KEGG analysis of 30 hub genes by KOBAS 3.0.

Subjects were categorized into low and high expression groups according to median CXCR4 expression level. Gene Ontology (GO) analysis showed that differential expression genes (DEGs) in subjects with higher CXCR4 expression were enriched in biological process (BP): regulation of RNA splicing, epigenetic modification, and mRNA export from the nucleus, etc.; cellular component (CC): histone deacetylase complex, high-density lipoprotein particle, etc.; and molecular function (MF): methylated histone binding, insulin receptor binding, etc. (Figures 2(a)–2(c)). Analysis of C2 curated gene sets (Table S2) exhibited that these genes were significantly enriched in signaling pathways such as the JNK-MAPK pathway, PI3K-AKT, mTOR signaling pathway, B-cell receptor (BCR) pathway, B-cell poor survival, IL-2 STAT5, IL-2 PI3k pathway, and NOTCH1 signal pathway. And genes had higher transcriptional activity of smad2/3/4 heterotrimer, adipogenesis, insulin resistance, VH rearrangement, doxorubicin resistance and alkylating resistance, CD5 target up, MYC and TFRC-up, and TP53 expression and degradation (Figure 2(d)). However, no gene sets were significantly enriched in the CXCR4 low group at FDR < 25%.

Based on the STRING database (https://string-db.org/), the PPI network of CXCR4 was constructed with a minimum required interaction score of 0.9. We selected 30 hub genes (C3, CCR7, AURKB, CDC45, CDC6, GNG7, S1PR1, CENPM, CX3CR1, EXO1, KIF4A, MCM10, ADRA2A, CCL21, CNR1, CXCL13, DRD4, GPR183, HRAS, PNOC, FOXM1, HJURP, SYK, CDT1, SELL, UQCR10, CDCA5, HIST1H2BD, HNRNPA2B1, HNRNPH1) as center nodes of the interaction network (Figure 2(e)). These hub genes were further analyzed using the online tool KOBAS 3.0 (http://kobas.cbi.pku.edu.cn/kobas3) to verify the results of GSEA (Figure 2(f)).

### 3.3. ICI and Gene Correlation Analysis by TIMER2.0

**Figure 3:**
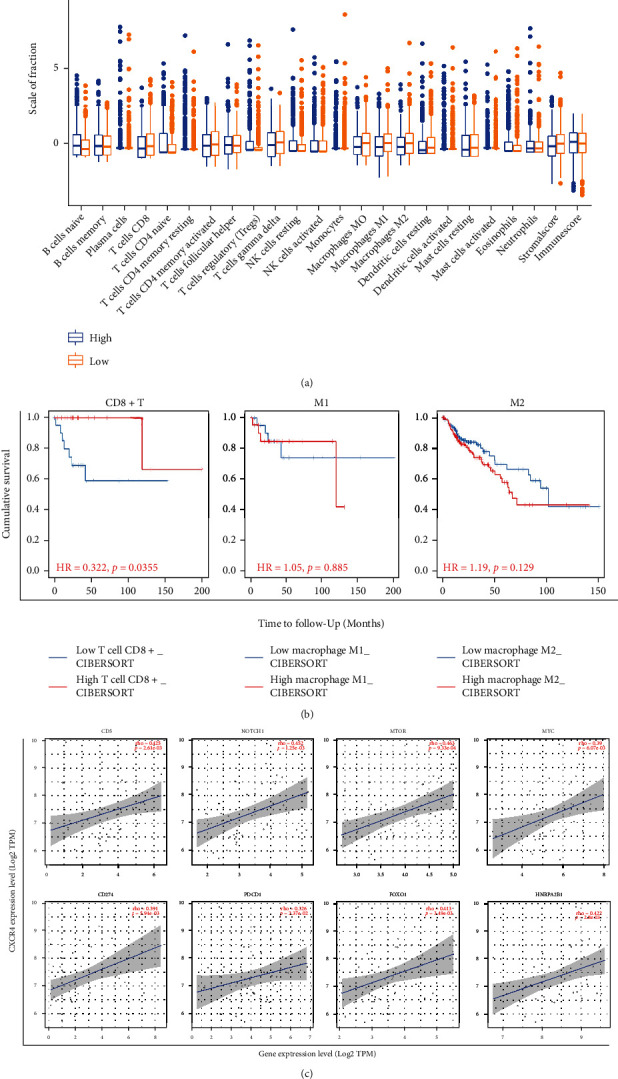
Immune cell infiltration and gene correlation analysis. (a) Immunocyte infiltration levels of 22 types of immune cells between high and low CXCR4 expression groups. (b) Relationship between degree of immune cell CD8 + T cell (left), macrophage 1 (middle) and macrophage 2 (right) infiltration, and DLBCL prognosis (CIBERSORT analytic module) by Kaplan-Meier plots (based on TCGA database). (c) TIMER2.0 analysis of gene correlation between CXCR4 and CD5, Notch1, MTOR, MYC, PDCD1 (PD-1), CD274 (PD-L1), FOXO1, and hnRNPA2B1.

The CIBERSORT algorithm was used to calculate the proportion of immune cells in 816 samples from databases of GPL570 (GSE11318, GSE23501, GSE19246, GSE31312, GSE53786, GSE64555, GSE56313, GSE74266, and GSE93986). We found that high CXCR4 expression tumor tissues harbored a higher level of naive B-cells (*P* < 0.01), naive CD4+ T cells (*P* < 0.01), resting memory CD4+ T cells (*P* < 0.05), and regulatory T cells (Tregs) (*P* < 0.05), while low CXCR4 expression group harbored a higher level of CD8+ T cells (*P* < 0.05), macrophage 1 (*P* < 0.001), macrophage 2 (*P* < 0.01), and resting dendritic cells (*P* < 0.05) than high expression ones **(**Figure 3(a)). The TIME2.0 website integrates a series of algorithms with TCGA database or the tumor expression data submitted by users themselves to estimate specific immune cell types. The run results are more reliable, and the results are displayed visually after a comprehensive evaluation through complex calculation methods. We used Kaplan-Meier plots to explore the relationship between ICI and DLBCL prognosis (CIBERSORT analytic module). We found that CD8 + T cell (*P* = 0.0355) infiltration was significantly correlated with DLBCL prognosis. There was no significant correlation between prognosis and macrophage 1 and macrophage 2 infiltration (Figure 3(b)). Based on the above GSEA and PPI results, we assessed the relationship between CXCR4 expression level and the expression of MYC, PDCD1(PD-1), CD274(PD-L1), Notch1, mTOR, CD5, FOXO1, and hnRNPA2 gene markers that showed significance in the GSEA analysis above using TIMER 2.0 (TCGA database). The results showed that the expression of CXCR4 was positively correlated with the expression of these genes (Figure 3(c)).

### 3.4. IHC Validation of CXCR4 Expression in DLBCL Patient Samples Associated with Clinicopathological Features

**Figure 4:**
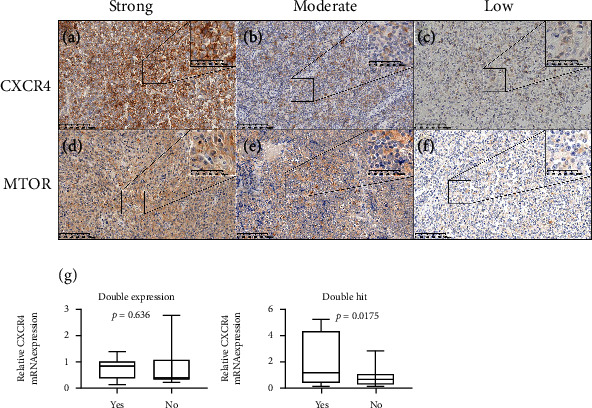
Representative photomicrographs (original magnification: ×200; upper right corner, ×400) of CXCR4 and MTOR immunohistochemical staining in DLBCL tissues. (a) Strongly positive expression status, (b) moderately positive expression status, and (c) weakly positive expression status of CXCR4 in DLBCL tissues. (d) Strongly positive expression status, (e) moderately positive expression status, and (f) weakly positive expression status of MTOR in DLBCL tissues. (g) Correlation between mRNA level of CXCR4 in DLBCL and prognostic characteristics of double expression (left) and double hit (right). High CXCR4 mRNA level was associated with double-hit DLBCL (*P* = 0.0175).

Tissue microarrays of 44 de novo DLBCL were examined by IHC, including 26 men and 18 women with a median age of 60 years (range, 29-82 years). Thirteen (29.5%) samples were positive for CXCR4, which were stained dark or light brown, and distributed in the membrane and cytoplasm of lymphoma cells (Figures 4(a)–4(c)). The clinicopathological features of the patients and their relationship with CXCR4 protein expression are summarized in Table 2. No association was identified between the expression of CXCR4 and age, gender, Ann Arbor stage, and IPI score (*P* > 0.05). The protein expression level of CXCR4 was found to be positively associated with the cell of origin (COO) of the non-GCB subtype (*P* = 0.022), and with high levels of the mTOR expression (Figures 4(d)–4(f)). No correlation was found between the expression level of CXCR4 and CD8 + T lymphocyte infiltration, c-MYC, tp53, and PD-1 and PD-L1 expression.

Further, to verify the mRNA level of CXCR4 in DLBCL and its correlation to clinical characteristics, another independent cohort of 59 de novo DLBCL patients, all diagnosed within 2 years and with complete treatment information, was applied for qPCR using their FFPE samples. Based on the qPCR results, high CXCR4 mRNA level was correlated to double-hit DLBCL (*P* = 0.0175) (Figure 4(g)). However, no correlation was found between the mRNA level of CXCR4 and double-expression DLBCL and Bruton's tyrosine kinase (BTK) inhibitor or lenalidomide treatment response.

### 3.5. Growth Inhibition Effect of CXCR4 Inhibitor WZ811 on DLBCL Cell Lines

**Figure 5:**
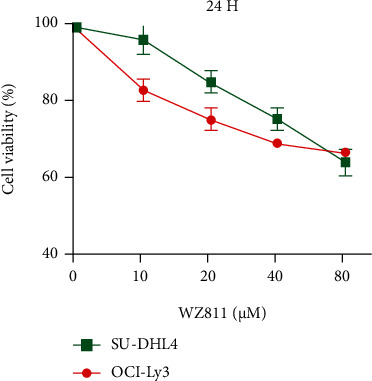
WZ811 inhibited the proliferation of the cell lines in a dose-dependent manner. DLBCL cells (OCI-Ly3 and SU-DHL4) were treated with indicated concentrations of WZ811 (0, 10, 20, 40, 80 *μ*M) for 24 h, and cell viability was measured by cell counting kit-8 assay.

With the escalation of drug concentration (10, 20, 40, 80 *μ*M), WZ811 inhibited the proliferation of DLBCL cell lines in a dose-dependent manner (Tables 3 and 4). OCI-ly3 cells exhibited more sensitivity to WZ811 treatment than SU-DHL4 cells within the range <80 *μ*M (Figure 5). However, according to IC50 results, with the escalation of WZ811 concentration, SU-DHL4 cells exhibited more sensitivity to WZ811 treatment than OCI-Ly3 cells with a 50% inhibitory concentration (IC50) of 372.2 *μ*M for OCI-Ly3 and 143.5 *μ*M for SU-DHL4. Analysis results of IC50 were achieved by CompuSyn software (Version 1.0). With the prolongation of treatment time, no statistical significance was found between 0 h, 2 h, and 24 h (data not shown).

## 4. Discussion

Although the standard immunochemotherapy of R-CHOP has cured a majority of patients with DLBCL, treatment failure after R-CHOP still remains an intractable problem due to worse outcomes, which further underlines the importance of finding novel targets. Alizadeh et al. established the COO classification of DLBCL by using cDNA microarrays for gene expression profiling (GEP): the GCB and ABC subtype [17]. DLBCL patients with ABC subtype showed significantly worse prognosis compared with those with GCB subtype [18]. In our meta-analysis, CXCR4 was highly expressed in DLBCL patients with the ABC subtype and this was related to poor OS (Figures 1(a) and 1(b)). Our GSEA results revealed that the tumorigenicity of CXCR4 in DLBCL is related to the PI3K/AKT/mitogen-activated protein kinase (MAPK) pathways, mTOR, JNK-MAPK signal transducer and activator of transcription (STAT) pathways, Smad2/3/4, NOTCH1, and WNT signal pathways (Figure 2(d)) [19, 20]. PI3K/AKT/mTOR pathway involved in crucial functions such as cellular proliferation, cell cycle regulation, and cell motility in DLBCL [21]. JAK-STAT signaling pathways were associated with pathogenesis of ABC-DLBCL [22]. DLBCL patients with the NOTCH1 mutations have worse PFS and OS [23]. These results are consistent with previous studies showing that the CXCR4/CXCL12 axis can mediate tumorigenicity by activating various intracellular signaling transduction pathways and downstream effectors [24–28]. Gene correlation analysis (TIMER) demonstrated that the CXCR4 expression is positively related to the degradation of tumor suppressor geneTP53, upregulation of the protooncogene C-MYC, and CD5expression (Figure 3(c)). CD5^+^ DLBCL has a poor prognosis and is classified as ABC-DLBCL with elusive genetic features [29]. This confirmed the role of CXCR4 in the ABC but not the GCB subtype of DLBCL, suggesting that CXCR4 exerts an adverse effect on the prognosis of the ABC subtype DLBCL through the above molecular signaling pathways. According to the study of Breinholt et al. [30], large B-cell lymphoma patient samples with significantly higher prevalence of MYC-BCL2-double-hit showed significantly lower expression of PD-L1 and tumor-associated macrophages. Meanwhile, our qPCR analysis showed that the high CXCR4 expression correlated to molecular cytogenetic abnormality of double-hit DLBCL **(**Figure 4(g)). The intrinsic relationship between CXCR4 and double-hit DLBCL deserves worth further research. However, we did not find correlations between the protein expression level of CXCR4 and CD8^+^T lymphocyte infiltration, CD5, c-MYC, tp53, and PD-1 and PD-L1 expression. This discrepancy may due to the limited sample size of the IHC cohort.

Immune cells in the tumor microenvironment contribute to tumor progression and the antitumor responses. Their biological significance in DLBCL varies from different studies. CD8^+^ T lymphocytes and M1 macrophage play an important role in antitumor immune responses. Our results showed that the low CXCR4 expression group harbored a higher level of CD8^+^ T cells (*P* < 0.05) (Figure 3(a)) and M1 macrophage (*P* < 0.001) (Figure S1), which may explain a better survival result of low CXCR4 expression group. One study assessed the safety, efficacy, and immunobiological effects of the CXCR4 antagonist (BL-8040, motixafortide) combined with a PD-1 inhibitor and chemotherapy in metastatic pancreatic ductal adenocarcinoma (PDAC) [10]. CXCR4 blockade promotes CD8^+^ effector T cell tumor infiltration and is synergistic with PD-1 inhibitors in PDAC mouse models. This study is in line with our study results. Besides, the high CXCR4 expression in DLBCL harbored a higher level of regulatory T cells (Tregs) (*P* < 0.05). It is known that Tregs are important modulators for the interaction between lymphoma cells and tumor microenvironment. Zhou [31] reported that a high infiltration of FOXP3/CTLA-4 double-positive cells was significantly associated with poor prognosis. Chang [32] found that higher infiltration of intr-tumoral CD25^+^ FOXP3^+^ lymphocytes correlate with a favorable prognosis in patients with DLBCL. Previous studies on the biological significance of immune cells infiltration in DLBCL [33, 34] are inconsistent. This may be due to the sample size and different materials (peripheral blood, tissue, and bone marrow) and methods (immunohistochemistry, flow cytometry, and sequencing). The effects of ICI in DLBCL on the development of DLBCL need to be verified by more fundamental experiments.

According to our GSEA results, CXCR4 coexpression genes are enriched during metabolism biological processes of adipogenesis and insulin resistance (Table S2). Recently, numerous studies have underlined the role of cholesterol metabolism in the tumorigenesis. Cholesterol metabolism regulates oncogenic signaling pathways, including the PTEN/AKT/mTORC1 [35–37], MYC [38], Wnt [39], MAPK [40], and Hippo pathway [41]. Rinks et al. [42] reduced cellular cholesterol by blocking the high-density lipoprotein nanoparticles (HDL NP), which can also block the enhanced BCR signaling. In their study, they achieved both cellular cholesterol reduction and apoptosis in resistant ABC DLBCL cell lines by using ibrutinib and the spleen tyrosine kinase inhibitor together with HDL NP. Thus, some new cholesterol metabolites have recently become promising target drugs for cancer treatment [43–45]. In another study, metformin, a drug that can enhance insulin sensitivity, was identified to be associated with improved response rate and PFS in diabetic patients and is a possible therapeutic drug against DLBCL [46]. These results illustrate that reduction of cellular cholesterol or restoration of insulin sensitivity in lymphoma is a new method to induce tumor cell apoptosis. Our combined GSEA results indicate that cholesterol metabolism and insulin resistance may regulate complex signaling pathways and antitumor immunity and play important roles in high CXCR4 expression DLBCL. We hypothesize that CXCR4 antagonists may simultaneously inhibit MAPK, PI3k/AKT, and mTOR signaling pathway, inhibit cholesterol metabolism, and correct insulin resistance, which may exert a stronger inhibitory effect on the BCR signaling pathway.

**Figure 6:**
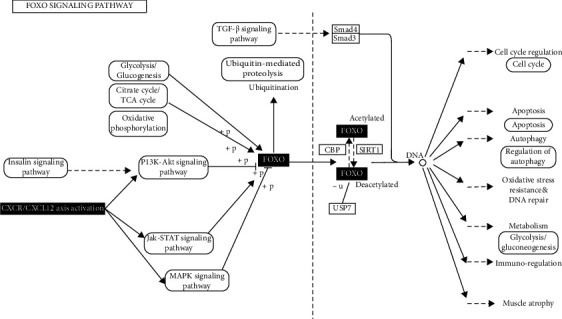
The forkhead box O (FOXO) protein family regulates the expression of genes in cellular physiological events including apoptosis, tumor angiogenesis, glucose metabolism, oxidative stress resistance, and DNA damage repair. PI3K-Akt signaling pathway is activated in response to CXCR4/CXCL12 activation. Then, FOXO proteins are phosphorylation by the serine/threonine kinase (AKT)/protein kinase B (PKB). Phosphorylated FOXOs result in the export of FOXOs from the nucleus to the cytoplasm, thereby decreasing expression of FOXO target genes. Other posttranslational modifications of FOXO include methylation, acetylation, ubiquitylation, and glycosylation.

Our GSEA results showed that genes from DLBCL patients with high CXCR4 expression are enriched in the forkhead box O- (FOXO-) mediated transcription of cell cycle genes. KOBAS analysis showed increased hub genes associated with the FOXO signaling pathway. FOXOs are tumor suppressors and can be used as key regulators of cell biology [47]. FOXO3a belongs to this family and functions as a trigger for apoptosis through the expression of genes necessary for cell death, and its inactivation is essential for the proliferation of immune cells. According to the study results of Kapoor et al. [48], Ibrutinib-resistant DLBCL (IB-R) cell lines showed downregulation of FOXO3a and PTEN levels in the nuclei and activation of AKT signaling. Therefore, they used a PI3K inhibitor and AKT inhibitor (idelalisib and MK2206) in IB-R cells, which resulted in increased ibrutinib-induced apoptosis. The drug selinexor (an exportin 1 inhibitor) together with ibrutinib can also increase the nuclear abundance of FOXO3a and PTEN in IB-R cells, leading to the restoration of ibrutinib-induced apoptosis. Our hub gene analysis revealed the coexpression of CXCR4 and hnRNPA2B1, which was confirmed by the (TIMER) gene correlation analysis. HnRNPA2B1 is involved in carcinogenesis by interacting with several proteins. One study found that it promotes the survival of tumor cells by preventing the activation of p53 [49]. In another study by Pan et al., CXCR4 was coimmunoprecipitated with cyclophilin A (CyPA). Stimulation of CXCR4 induces CyPA phosphorylation and nuclear translocation. CyPA formed a complex with heterogeneous nuclear ribonucleoprotein (hnRNP) A2, which underwent nuclear export in response to the activation of CXCR4 and was blocked by RNAi of CyPA. Overall, these findings demonstrate that the CXCR4 antagonist exerts both effects on BCR signaling and nuclear export, restores the function of FOXO3a (Figure 6) and PTEN, reduces the nuclear export of hnRNPA2, and overcomes acquired resistance to BTK inhibitor in DLBCL patients.

According to our cell viability test, WZ811 suppressed the cell viability of both ABC-DLBCL (OCI-Ly3) and GCB-DLBCL (SU-DHL4), which confirmed the results of our bioinformatics analysis (Figure 5). Currently, there are a rising number of clinical trials using CXCR4 antagonists for treating different malignant diseases [50]. One phase I study (NCT00903968) enrolled relapse/refractory multiple myeloma (RRMM). The study drugs were bortezomib (1.3 mg/m^2^) and CXCR4 antagonist (the maximal-tolerated dose 0.32 mg/kg). Results of this clinical trial indicated that combination of these two drugs is safe and effective in RRMM. A phase Ib/II study also aimed to determine the safety and tolerability of the anti-CXCR4 antibody (ulocuplumab), alone and in combination with lenalidomide and dexamethasone, or with bortezomib and dexamethasone in patients with RRMM. This study showed that ulocuplumab is safe with acceptable adverse events and leads to a high response rate in combination with lenalidomide and dexamethasone in patients with RRMM. These results suggest that CXCR4 inhibitors are promising antimyeloma drugs. However, few clinical trials have been conducted on DLBCL; so, there is a potential for further exploration in clinical trials.

Certain limitations existed in our study. For example, there were a small number of verification samples in this study. Additionally, we simply divided the expression level of CXCR4 into high and low groups according to the median CXCR4 expression level and used negative and positive in the immunohistochemical stains. However, choosing the best immunohistochemical cutoff value may help us more sensitively and specifically identify high-risk DLBCL patients with the positive CXCR4 expression. Also, we used FFPE samples for qPCR due to lacking of fresh biopsy specimen, which may reduce the accuracy of experimental results. Limited variety of cell lines was also the shortcoming of our experiment. We hope to make up for the defects of these experiments in the future.

## 5. Conclusion

Overall, our study utilized a comprehensive analysis with experimental validation to imply the therapeutic significance of CXCR4 as a potential target in DLBCL, which may pave the way for precision therapy and translational medicine in the prognosis and treatment of DLBCL in the future.

## Figures and Tables

**Table 1:** Information of the included GEO datasets.

	Year	GEO accession	Platform	Country	Patient number	Clinicopathological parameters
1	2006	GSE4732	GPL3706	USA	216	Subtype, gender, age, stage, OS
2	2008	GSE11318	GPL570	USA	172	Subtype, gender, age, stage, OS
3	2009	GSE16920	GPL7015	Japan	46	Gender, age
4	2010	GSE23501	GPL570	USA	69	Subtype, gender, age, OS, PFS
5	2010	GSE19246	GPL570	USA	59	Subtype
6	2011	GSE22470	GPL96	Germany	271	Subtype, gender, age
7	2012	GSE31312	GPL570	USA	498	Subtype
8	2012	GSE32918	GPL8432	UK	172	Subtype, gender, age, OS
9	2012	GSE38202	GPL8432	South Korea	164	Gender, age
10	2013	GSE53786	GPL570	USA	119	Subtype, gender, age, stage, OS
11	2013	GSE44164	GPL96	Germany	32	Subtype, gender, age
12	2014	GSE64555	GPL570	UK	40	Subtype
13	2014	GSE57612	GPL96	Germany	37	Subtype, gender, age, stage, OS
14	2015	GSE69051	GPL14951	UK	160	Age, OS
15	2015	GSE69049	GLP8432	UK	32	Age, OS
16	2015	GSE56313	GPL570	Denmark	55	Subtype
17	2016	GSE74266	GPL570	Denmark	62	Subtype
18	2017	GSE93986	GPL570	USA	88	Subtype, PFS
19	2017	GSE87371	GPL570	France	223	Age, gender, stage, PFS, OS

Abbreviations: GEO: Gene Expression Omnibus; OS: overall survival; PFS: progression-free survival.

**Table 2:** Relationship between CXCR4 expression status and clinicopathological features in DLBCL.

		CXCR4 negative	CXCR4 positive	*P* value
Age	<60 ≥60	16 15	5 8	0.426

Gender	Male Female	17 14	9 4	0.376

Ann Arbor stage	I-II III-IV	17 14	5 8	0.322

COO	GCB Non-GCB	25 6	6 7	0.022

IPI score	0-1 ≥2	9 22	3 10	0.686

MTOR	<20% ≥20%	21 10	4 9	0.024

CD5	<50% ≥50%	20 11	9 4	0.763

CD8 + T cell infiltration	<20% ≥20%	14 17	8 5	0.322

C-MYC	<50% ≥50%	15 16	8 5	0.426

TP53	<50% ≥50%	21 10	6 7	0.180

PD-1	≤1% >1%	4 27	3 10	0.404

PD-L1	≤1% >1%	22 9	10 3	0.686

Abbreviations: COO: cell of origin.

**Table 3:** Cell viability of OCI-Ly3 and SU-DHL4 treated with WZ811.

WZ811 concentration (*μ*M)		Cell viability %		
OCI-Ly3	0 (group 1)	100	100	100
	10 (group 2)	79.99	86.18	83.95
	20 (group 3)	77.87	76.81	72.07
	40 (group 4)	68.54	68.69	70.56
	80 (group 5)	67.45	67.62	66.15

SU-DHL4	0 (group 6)	100	100	100
	10 (group 7)	98.75	91.94	98.8
	20 (group 8)	83.47	88.53	84.29
	40 (group 9)	73.31	78.82	75.12
	80 (group 10)	61.4	63.52	68.27

**Table 4:** Statistical results of cell viability test.

Bonferroni's multiple comparison test		*P* value
OCI-Ly3	Group 2 vs. group 1	*P* < 0.0001
	Group 3 vs. group 1	*P* < 0.0001
	Group 4 vs. group 1	*P* < 0.0001
	Group 5 vs. group 1	*P* < 0.0001
	Group 3 vs. group 2	NS
	Group 4 vs. group 2	*P* < 0.0001
	Group 5 vs. group 2	*P* < 0.0001
	Group 4 vs. group 3	NS
	Group 5 vs. group 3	*P* < 0.05
	Group 5 vs. group 4	NS
	Group 6 vs. group 7	NS
	Group 6 vs. group 8	*P* < 0.0001
	Group 6 vs. group 9	*P* < 0.0001
	Group 6 vs. group 10	*P* < 0.0001

SU-DHL4	Group 7 vs. group 8	*P* < 0.001
	Group 7 vs. group 9	*P* < 0.0001
	Group 7 vs. group 10	*P* < 0.0001
	Group 8 vs. group 9	*P* < 0.005
	Group 8 vs. group 10	*P* < 0.0001
	Group 9 vs. group 10	*P* < 0.001

## Data Availability

The data used to support the findings of this study are included within the article.

## Abbreviations

EZH2: Enhancer of zeste homolog 2
PI3K: Phosphatidylinositol-3-kinase
mTOR: Mammalian target of rapamycin
CXCR4: CXC chemokinereceptor 4
TCGA: The Cancer Genome Atlas
GEO: Gene Expression Omnibus
GSEA: Gene set enrichment analysis
PPI: Protein-protein interaction
TIMER: Tumor Immune Estimate Resource
ICI: Immune cell infiltration.

## Supplementary Materials

Supplementary Materials Supplementary Tables. Table S1: list of antibodies. Table S2: results of gene set enrichment analysis (c2.all.v7.2.symbols.gmt). Supplementary Figure. Figure S1: relation between CXCR4 expression and macrophage M1 infiltration.Low CXCR4 expression group harbored a higher level of M1 macrophage infiltration (*P* < 0.001).
