# Supplementary material for: Identification of CXCR4 Upregulation in Diffuse Large B-Cell Lymphoma Associated with Prognostic Significance and Clinicopathological Characteristics
Source: Dis Markers. 2022 Jun 21;2022:3276925. doi: 10.1155/2022/3276925 (PMC9239773; doi:10.1155/2022/3276925)

**Supplementary Table**

Table S1 List of antibodies

| **Antigen** | **Clone** | **Dilution** |
| --- | --- | --- |
| CD5 | 4C7 | 1:200 |
| CD8 | 4B11 | 1:100 |
| PD1 | EPR4877 | 1:200 |
| PDL1 | 28-8 | 1:500 |
| P53 | DO-7 | 1:200 |
| C-MYC | Y69 | 1:50 |
| CXCR4 | UMB2 | 1:200 |
| MTOR | 7C10 | 1:100 |

Table S2 Results of Gene Set Enrichment Analysis (c2.all.v7.2.symbols.gmt)

| **Gene set name** | **NSE** | **NOM *p-*value** | **FDR *q*-value** |
| --- | --- | --- | --- |
| ST_JNK_MAPK_PATHWAY | 2.458465 | ＜0.001 | 0.003877606 |
| CHANDRAN_METASTASIS_UP | 2.2625566 | ＜0.001 | 0.02311522 |
| WP_B_CELL_RECEPTOR_SIGNALING_PATHWAY | 2.1655927 | 0.002012072 | 0.036347024 |
| MORI_MATURE_B_LYMPHOCYTE_UP | 2.1413798 | ＜0.001 | 0.033613194 |
| REACTOME_TRANSCRIPTIONAL_ACTIVITY_OF_SMAD2_SMAD3_SMAD4_HETEROTRIMER | 2.123015 | 0.001968504 | 0.03846271 |
| BIOCARTA_BCR_PATHWAY | 2.1008494 | ＜0.001 | 0.042326204 |
| BURTON_ADIPOGENESIS_12 | 2.0566397 | ＜0.001 | 0.049167603 |
| SARTIPY_NORMAL_AT_INSULIN_RESISTANCE_DN | 2.0541377 | 0.002008032 | 0.04901146 |
| WP_MRNA_PROCESSING | 2.0513418 | 0.025291828 | 0.048349682 |
| PID_IL2_STAT5_PATHWAY | 2.0500662 | 0.001926782 | 0.046727207 |
| BIOCARTA_FCER1_PATHWAY | 2.046385 | 0.004201681 | 0.04638207 |
| SIG_PIP3_SIGNALING_IN_B_LYMPHOCYTES | 2.0482316 | 0.00408998 | 0.046526007 |
| CHEN_HOXA5_TARGETS_9HR_UP | 2.0413435 | 0.00996016 | 0.047520015 |
| WP_PDGFRBETA_PATHWAY | 2.0134966 | 0.010141988 | 0.05968836 |
| PID_EPO_PATHWAY | 2.005575 | 0.00589391 | 0.062243767 |
| REACTOME_RUNX1_REGULATES_GENES_INVOLVED_IN_MEGAKARYOCYTE_DIFFERENTIATION_AND_PLATELET_FUNCTION | 1.9961978 | 0.001992032 | 0.06684018 |
| BIOCARTA_IGF1_PATHWAY | 1.98987 | 0.002070393 | 0.069428906 |
| ZHAN_MULTIPLE_MYELOMA_PR_DN | 1.988111 | 0.001953125 | 0.068008505 |
| KEGG_MTOR_SIGNALING_PATHWAY | 1.9734266 | 0.006072875 | 0.07520198 |
| FAELT_B_CLL_WITH_VH_REARRANGEMENTS_DN | 1.9618165 | 0.013833992 | 0.076613985 |
| REACTOME_DOWNREGULATION_OF_SMAD2_3_SMAD4_TRANSCRIPTIONAL_ACTIVITY | 1.951475 | 0.008064516 | 0.079099104 |
| BIOCARTA_IL2_PATHWAY | 1.9393641 | 0.007905139 | 0.08167012 |
| PID_IL2_PI3K_PATHWAY | 1.9298497 | 0.008097166 | 0.08614047 |
| KANG_DOXORUBICIN_RESISTANCE_DN | 1.8788862 | 0.001937985 | 0.11037204 |
| BACOLOD_RESISTANCE_TO_ALKYLATING_AGENTS_DN | 1.8567839 | 0.001980198 | 0.1256161 |
| HUTTMANN_B_CLL_POOR_SURVIVAL_DN | 1.8301789 | 0.013565891 | 0.13381088 |
| REACTOME_ONCOGENE_INDUCED_SENESCENCE | 1.8270249 | 0.010141988 | 0.1357721 |
| GUTIERREZ_CHRONIC_LYMPHOCYTIC_LEUKEMIA_DN | 1.8214905 | 0.012578616 | 0.14069076 |
| PID_PI3KCI_AKT_PATHWAY | 1.8201506 | 0.010351967 | 0.13855545 |
| BIOCARTA_GPCR_PATHWAY | 1.8069715 | 0.002012072 | 0.14513803 |
| BILBAN_B_CLL_LPL_DN | 1.7891959 | 0.032786883 | 0.14935684 |
| GARY_CD5_TARGETS_UP | 1.787876 | 0.014767933 | 0.14967576 |
| WINTER_HYPOXIA_DN | 1.7706432 | 0.02053388 | 0.15681292 |
| WINTER_HYPOXIA_DN | 1.7706432 | 0.02053388 | 0.15681292 |
| PID_MAPK_TRK_PATHWAY | 1.7670945 | 0.014373717 | 0.15769076 |
| WP_IL5_SIGNALING_PATHWAY | 1.7595562 | 0.030612245 | 0.16335292 |
| ODONNELL_TARGETS_OF_MYC_AND_TFRC_UP | 1.7535777 | 0.014705882 | 0.16852026 |
| BAKKER_FOXO3_TARGETS_UP | 1.7521403 | 0.023346303 | 0.16816267 |
| REACTOME_FOXO_MEDIATED_TRANSCRIPTION_OF_CELL_CYCLE_GENES | 1.7517776 | 0.022540983 | 0.16643105 |
| REACTOME_REGULATION_OF_TP53_EXPRESSION_AND_DEGRADATION | 1.7359546 | 0.026156941 | 0.18001904 |
| PID_MTOR_4PATHWAY | 1.7359291 | 0.022403259 | 0.17896104 |
| WP_MAPK_CASCADE | 1.7258834 | 0.020576132 | 0.18521808 |
| WP_PI3KAKTMTOR_SIGNALING_PATHWAY_AND_THERAPEUTIC_OPPORTUNITIES | 1.7197961 | 0.0332681 | 0.18867745 |
| REACTOME_SIGNALING_BY_NOTCH1 | 1.660821 | 0.0234375 | 0.23559207 |

**Supplementary figure legends**

Figure S1

Relation between CXCR4 and Macrophage M1


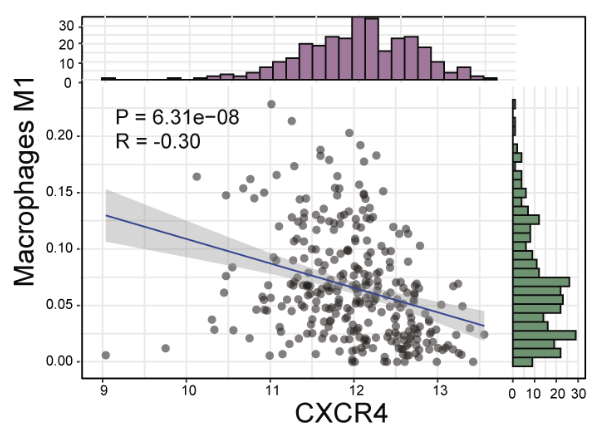

Supplement: Supplementary Materials — Supplementary Tables. Table S1: list of antibodies. Table S2: results of gene set enrichment analysis (c2.all.v7.2.symbols.gmt). Supplementary Figure. Figure S1: relation between CXCR4 expression and macrophage M1 infiltration.Low CXCR4 expression group harbored a higher level of M1 macrophage infiltration (P < 0.001). [file 3276925.f1.docx]
